# Association between single-point insulin sensitivity estimator and three-vessel coronary disease: a cross-sectional study

**DOI:** 10.3389/fendo.2026.1838548

**Published:** 2026-05-12

**Authors:** Wei Yang, Gaoying Dai, Nanhu Quan, Qian Tong

**Affiliations:** Department of Cardiovascular Center, The First Hospital of Jilin University, Changchun, China

**Keywords:** cross-sectional study, insulin resistance, single-point insulin sensitivity estimator, three-vessel coronary disease, triglyceride-glucose index

## Abstract

**Background:**

The single-point insulin sensitivity estimator (SPISE) is a novel surrogate marker for assessing insulin resistance in humans. Three-vessel coronary disease (TVD) remains a major challenge in coronary interventional therapy. However, the association between the SPISE index and TVD remains unclear. Therefore, this study aimed to evaluate the relationship between SPISE and TVD in an elderly population.

**Methods:**

Generalized additive models (GAM) and random forest variable importance scores were applied for feature selection to identify key predictors for constructing a TVD risk model. Multivariable regression and restricted cubic spline (RCS) analyses were performed to investigate the association between SPISE and TVD. Subgroup analyses were further conducted to assess potential effect modification.

**Results:**

GAM and random forest variable importance scores identified age and SPISE as the most important predictors. After adjustment for all covariates, SPISE was inversely associated with TVD (OR = 0.75, 95% CI: 0.68-0.83, *P* < 0.001). RCS and segmented regression analyses revealed a significant nonlinear relationship with a distinct inflection point. Receiver operating characteristic (ROC) analysis demonstrated that SPISE showed superior performance compared with the TyG index in predicting TVD risk (AUC: 0.718 vs. 0.701). Subgroup analyses confirmed a consistent inverse association between SPISE and TVD, with significant interactions observed across strata of sex, alcohol consumption, age, and BMI.

**Conclusion:**

A higher SPISE index is independently and inversely associated with TVD and may serve as a reliable predictor of disease risk, highlighting its potential utility in cardiovascular risk assessment.

## 1 Introduction

Three-vessel coronary disease (TVD) is defined as the presence of ≥50% angiographic stenosis in all three major coronary arteries, including the left anterior descending artery, left circumflex artery, and right coronary artery (1). As a distinct and severe form of coronary artery disease (CAD), patients with TVD are at substantially higher risk of adverse cardiovascular events (2). Given its strong association with increased morbidity and mortality, accurate identification and risk stratification of TVD are crucial for optimizing therapeutic strategies and improving clinical outcomes (3). Insulin resistance (IR), characterized by a reduced biological response to insulin-mediated glucose utilization, is a well-established risk factor for both microvascular and macrovascular complications (4), including metabolic syndrome (MetS), cardiovascular disease (CVD), and type 2 diabetes mellitus (5). IR contributes to both the initiation and progression of atherosclerosis and is closely linked to an increased risk of atherothrombotic cardiovascular events (6) Abnormal glucose metabolism and diabetes are associated with a greater atherosclerotic burden and a higher incidence of adverse cardiovascular outcomes (7, 8).

Although the hyperinsulinemic–euglycemic clamp (HEC) is considered the gold standard for assessing IR, its high cost and invasive nature limit its applicability in large-scale clinical studies (9, 10). The triglyceride–glucose (TyG) index has emerged as a reliable surrogate marker of IR and has been shown to be associated with the development and prognosis of CVD (11). Elevated TyG levels are significantly correlated with both the presence and severity of CAD (12). More recently, the single-point insulin sensitivity estimator (SPISE) has been proposed as a promising alternative marker of IR. Requiring only a single fasting blood sample, SPISE offers practical advantages for large-scale population screening to identify individuals at high cardiovascular risk (13). Higher SPISE values have been associated with a lower risk of future cardiovascular events in patients with type 2 diabetes (14). SPISE has emerged as a novel marker reflecting IR and has shown promising performance (15). Individuals classified as having IR exhibit significantly lower SPISE values, supporting its potential utility as a predictive indicator of IR (16). Furthermore, a cross-sectional study reported that SPISE was inversely associated with the prevalence of heart failure in older adults, with an odds ratio of 0.87 and a 95% confidence interval of 0.80 to 0.94 (*P* < 0.001) (17).

While the TyG index is a well-established surrogate for IR, combining more metabolic markers may improve cardiovascular risk prediction. As a novel composite index, SPISE is theoretically more comprehensive. However, no study has directly compared the performance of SPISE and TyG in predicting TVD among high-risk individuals. This study therefore performed a head-to-head comparison to evaluate whether SPISE offers incremental predictive value over the commonly used TyG index.

## 2 Materials and methods

### 2.1 Study population

**Figure 1:**
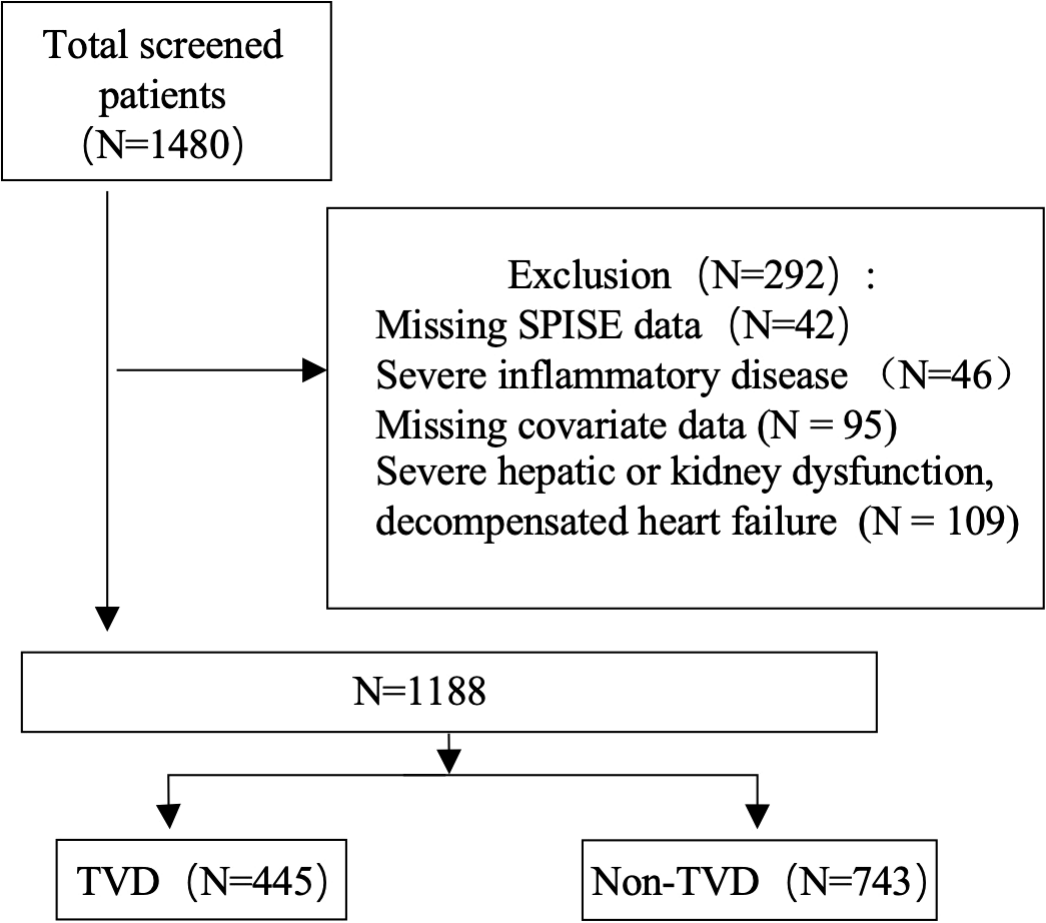
Flow chart.

In this retrospective study, we reviewed the clinical records of 1,480 hospitalized patients at the First Hospital of Jilin University between January 1, 2022 and December 31, 2025. After applying the inclusion and exclusion criteria, a total of 1,188 patients were ultimately included in the analysis, as illustrated in the flowchart in **Figure 1**. All participant data were anonymized during the data collection process. The main exclusion criteria included incomplete SPISE data, severe hepatic or renal dysfunction, decompensated heart failure, systemic inflammatory diseases and active infections requiring systemic treatment, and missing covariate information. Given the retrospective nature of the study, the requirement for informed consent was waived.

Collected data included demographic characteristics such as sex and age, anthropometric measurements including height and weight, medical history including hypertension, diabetes mellitus, smoking status, alcohol consumption, and prior myocardial infarction (MI), as well as laboratory parameters. These laboratory variables included white blood cell count (WBC), lymphocyte count (LY), serum creatinine (Cr), albumin (Alb), high-density lipoprotein cholesterol (HDL-C), low-density lipoprotein cholesterol (LDL-C), total cholesterol (TC), and triglycerides (TG), fasting blood glucose (FBG), uric acid (UA), left ventricular ejection fraction (LVEF), c−reactive protein (CRP), and glycated hemoglobin (HbA1c).

### 2.2 Definitions

According to established clinical guidelines, three-vessel disease was defined as the presence of at least 50% stenosis in all three major epicardial coronary arteries (18). Coronary angiography was used to assess the severity of coronary stenosis, and all images were independently evaluated by two experienced interventional cardiologists. Discrepancies were resolved through joint discussion to reach a consensus. Body Mass Index (BMI) was calculated as the ratio of body weight (kg) to squared height (meters).

SPISE = [600 × HDL-C (mg/dL)^0.185^]/[TG (mg/dL)^0.2^ × BMI (kg/m²)^1.338^] (19).

TyG = ln[TG(mg/dL)× FBG(mg/dL)/ 2] (20).

### 2.3 Statistical analysis

Continuous variables were expressed as mean with standard deviation or median with interquartile range, depending on data distribution, while categorical variables were presented as counts and percentages. Comparisons between the TVD and non-TVD groups were performed using the independent t test for normally distributed variables and the Mann–Whitney U test for non-normally distributed variables. Categorical variables were compared using the chi-square test. A two-sided P value less than 0.05 was considered statistically significant. All analyses were conducted using R software version 4.5.0. Random forest variable importance scores and generalized additive models (GAM) were used to identify important predictors and evaluate potential nonlinear associations. Three regression models were constructed. Model 1 was unadjusted. Model 2 was adjusted for age and sex. Model 3 was further adjusted for sex, age, hypertension, diabetes, MI, smoking, alcohol consumption, LVEF, Alb, FBG, UA, LY, Cr, CRP and HbA1c.

### 2.4 Sensitivity and robustness analysis

Variance inflation factors were used to assess multicollinearity. Generalized additive models and restricted cubic spline analyses were applied to validate potential nonlinear associations. Subgroup analyses were also performed to evaluate the robustness of the findings.

## 3 Results

### 3.1 Baseline characteristics

Among 1480 initially screened participants, the prevalence of TVD was 34.3%, compared with 37.5% in the final analytical cohort of 1188 individuals (**Table 1**). No significant between-group difference was observed (*P* > 0.05). Patients with TVD were older and had higher BMI, with a higher prevalence of hypertension, diabetes, and myocardial infarction (all *P* < 0.05). They also exhibited lower HDL-C, Alb, LY, and LVEF, but higher UA and Cr levels (all *P* < 0.05). LDL-C was slightly lower, while no significant differences were observed in TC, TG, FBG, WBC, CRP, or TyG. HbA1c was marginally higher in the TVD group. Notably, SPISE levels were significantly lower in patients with TVD (5.63 vs. 6.21, *P* < 0.001).

**Table 1:** Baseline characteristics.

Characteristic	Overall N = 1,188^1^	No N = 743^1^	Yes N = 445^1^	*p*-value^2^	SMD
Gender				0.400	0.052
*Female*	416 (35%)	267 (36%)	149 (33%)		
*Male*	772 (65%)	476 (64%)	296 (67%)		
Age (years)	64 (56, 71)	62 (55, 69)	68 (60, 73)	<0.001	0.477
Height (m)	1.68 (1.60, 1.72)	1.68 (1.60, 1.72)	1.68 (1.60, 1.72)	0.500	0.041
Weight (kg)	70 (61, 79)	70 (60, 77)	71 (63, 80)	<0.001	0.209
Smoking				0.800	0.018
*No*	802 (68%)	504 (68%)	298 (67%)		
*Yes*	386 (32%)	239 (32%)	147 (33%)		
Drinking				0.300	0.069
*No*	1,007 (85%)	623 (84%)	384 (86%)		
*Yes*	181 (15%)	120 (16%)	61 (14%)		
Hypertension				0.032	0.129
*No*	493 (41%)	326 (44%)	167 (38%)		
*Yes*	695 (59%)	417 (56%)	278 (62%)		
Diabetes				<0.001	0.261
*No*	814 (69%)	543 (73%)	271 (61%)		
*Yes*	374 (31%)	200 (27%)	174 (39%)		
BMI	25.0 (22.9, 27.3)	24.5 (22.8, 26.8)	26.0 (23.5, 27.9)	<0.001	0.301
LVEF (%)	61 (56, 63)	62 (56, 64)	60 (55, 63)	0.006	0.137
MI				0.001	0.191
*No*	700 (59%)	464 (62%)	236 (53%)		
*Yes*	488 (41%)	279 (38%)	209 (47%)		
WBC	7.36 (6.14, 8.90)	7.31 (6.08, 9.02)	7.45 (6.21, 8.74)	>0.900	0.039
LY	1.85 (1.39, 2.37)	1.89 (1.42, 2.42)	1.74 (1.34, 2.27)	0.010	0.121
Alb	39.1 (36.8, 41.8)	39.3 (37.2, 42.0)	38.5 (36.6, 41.3)	<0.001	0.181
UA	332 (267, 398)	324 (262, 384)	351 (280, 420)	<0.001	0.251
Cr	68 (56, 81)	66 (54, 79)	70 (58, 87)	<0.001	0.282
TC (mg/dl)	160 (131, 195)	162 (132, 197)	155 (128, 195)	0.120	0.072
TG (mg/dl)	131 (93, 190)	125 (90, 187)	136 (96, 193)	0.100	0.056
HDL (mg/dl)	39 (34, 46)	40 (34, 46)	38 (32, 44)	<0.001	0.151
LDL (mg/dl)	101 (78, 128)	103 (79, 130)	97 (77, 125)	0.046	0.110
FBG (mg/dl)	106 (92, 137)	106 (92, 135)	107 (92, 139)	0.900	0.095
HbA1c (%)	6.00 (5.60, 6.40)	5.90 (5.60, 6.40)	6.00 (5.70, 6.60)	0.027	0.099
SPISE	5.97 (5.12, 7.02)	6.21 (5.29, 7.14)	5.63 (4.93, 6.68)	<0.001	0.275
TyG	8.92 (8.48, 9.42)	8.88 (8.45, 9.38)	8.97 (8.54, 9.43)	0.130	0.095
CRP	1.68 (0.88, 3.14)	1.69 (0.87, 3.17)	1.68 (0.92, 3.14)	0.700	0.002

^1^ n (%); Median (Q1, Q3).

^2^ Pearson’s Chi-squared test; Wilcoxon rank sum test.

BMI, body mass index; HDL, high-density lipoprotein; LDL, low-density lipoprotein; UA, uric acid; HbA1c, glycosylated hemoglobin; TG, triglyceride; FBG, fasting blood glucose; MI, myocardial infarction; LVEF, left ventricular ejection fraction; Alb, albumin; WBC, white blood cell; LY, lymphocyte count; Cr, creatinine. SPISE, single-point insulin sensitivity estimator; TyG, triglyceride−glucose index; CRP, C−reactive protein; SMD, standardized mean difference.

SPISE values were evenly distributed across deciles with similar sample sizes in each group (n = 118–119). Variability differed by subgroup: the lowest SPISE decile showed greater dispersion (SD = 0.446), whereas mid-to-high deciles were more stable (SD = 0.10–0.15). TVD events were more frequent at lower SPISE deciles and decreased at higher levels, suggesting a nonlinear relationship.

### 3.2 Screening and validation of SPISE

**Figure 2:**
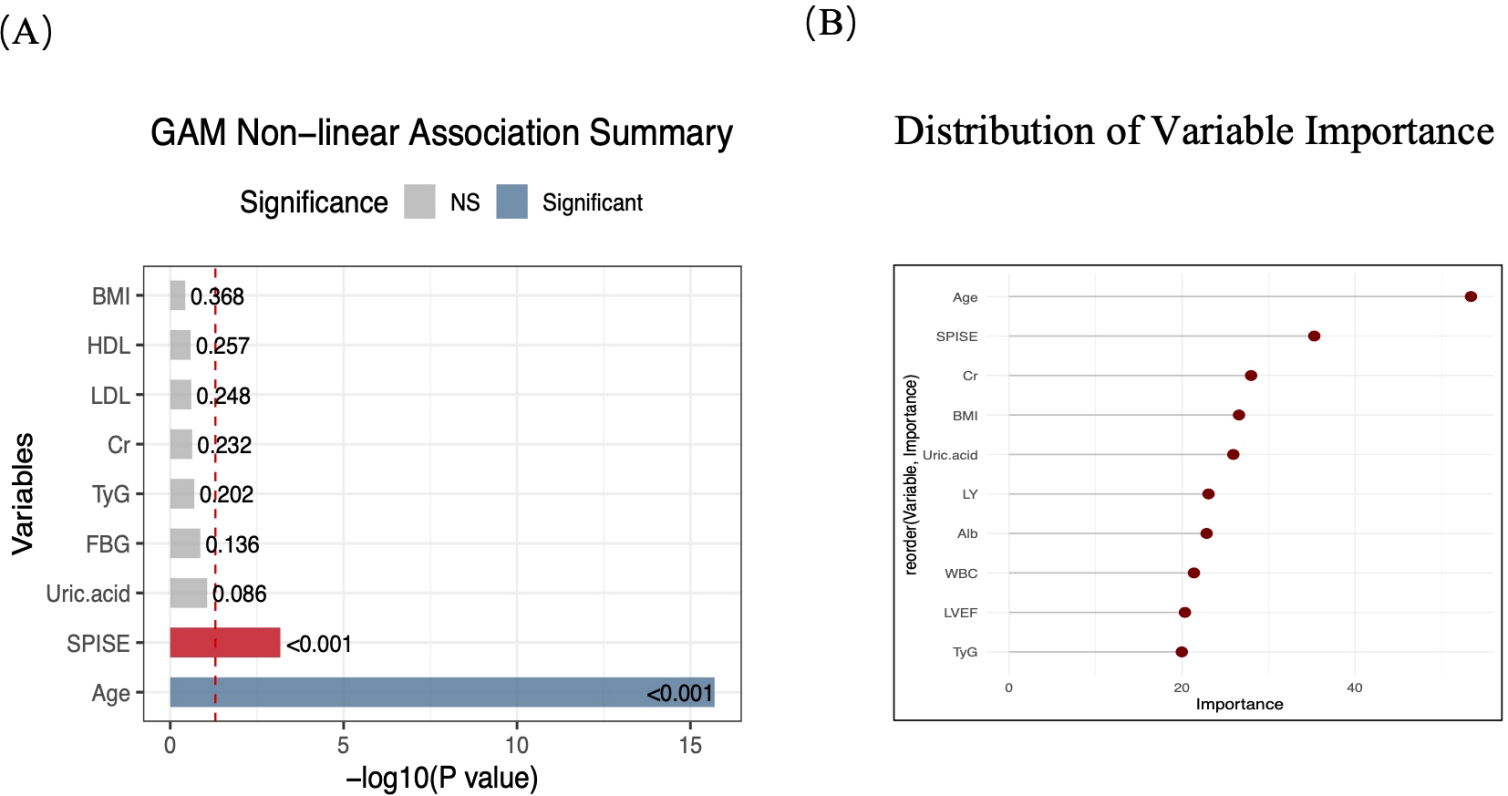
Generalized additive model and random forest for predicting TVD. **(A)** Generalized Additive Model analysis. **(B)** Variable importance based on the Random Forest model. TVD, three-vessel coronary disease; SPISE, single-point insulin sensitivity estimator.

As shown in **Figure 2**, age and SPISE were all significantly associated with the outcome in a nonlinear manner (*P* < 0.05). Random forest variable importance scores were subsequently performed to identify the most important predictors of the outcome. The variable importance ranking showed that age had the highest importance score (53.39), followed by SPISE (35.29), Cr (27.98), BMI (26.58). Among these variables, age emerged as the strongest predictor, with SPISE ranking second, indicating its substantial contribution to TVD. When considered together with the findings from the generalized additive models, these results consistently highlight SPISE as a key variable.

### 3.3 Regression analysis

Multivariable logistic regression analyses were performed to evaluate the association between SPISE and the risk of TVD across three models (**Table 2**). When analyzed as a continuous variable, higher SPISE levels were consistently associated with a lower risk of TVD in all models. Specifically, the odds ratios were 0.83 (95% CI: 0.76-0.90, *P* < 0.001) in Model 1, 0.73 (95% CI: 0.66-0.80, *P* < 0.001) in Model 2, and 0.75 (95% CI: 0.68-0.83, *P* < 0.001) in Model 3. When SPISE was categorized into quartiles, a clear decreasing trend in TVD risk was observed with increasing SPISE levels. Compared with the lowest quartile, the association became more pronounced after adjustment for confounders. In the fully adjusted Model 3, the odds ratios for the second, third, and fourth quartiles were 0.63 (95% CI: 0.44-0.90, *P* = 0.012), 0.20 (95% CI: 0.13-0.30, *P* < 0.001), and 0.30 (95% CI: 0.20-0.45, *P* < 0.001), respectively. Notably, the protective association was strongest in the third quartile, while a slight attenuation was observed in the highest quartile. Overall, these findings indicate that higher SPISE levels are independently associated with a reduced risk of TVD, and the consistency across models supports the robustness of this relationship.

**Table 2:** Logistics regression analysis of SPISE with TVD.

		Model 1		Model 2		Model 3	
Group		OR (95% CI)	*p*-value	OR (95% CI)	*p*-value	OR (95% CI)	*p*-value
SPISE Continuous	SPISE	0.83 (0.76, 0.90)	<0.001	0.73 (0.66, 0.80)	<0.001	0.75 (0.68, 0.83)	<0.001
SPISE Categorical							
	Q1	—		—		—	
	Q2	0.76 (0.55, 1.05)	0.100	0.60 (0.42, 0.85)	0.004	0.63 (0.44, 0.90)	0.012
	Q3	0.31 (0.22, 0.44)	<0.001	0.19 (0.13, 0.28)	<0.001	0.20 (0.13, 0.30)	<0.001
	Q4	0.46 (0.33, 0.64)	<0.001	0.27 (0.19, 0.39)	<0.001	0.30 (0.20, 0.45)	<0.001

CI, Confidence Interval; OR, Odds Ratio.

Model 1, unadjusted. Model 2, adjusted for age, sex. Model 3, adjusted for sex, age, smoking, alcohol consumption, hypertension, diabetes mellitus, myocardial infarction, serum creatinine, albumin, fasting blood glucose, uric acid, left ventricular ejection fraction, lymphocyte count, c−reactive protein, glycated hemoglobin. 95% CI, 95% confidence interval; OR, odds ratio.

### 3.4 ROC curve analysis

**Figure 3:**
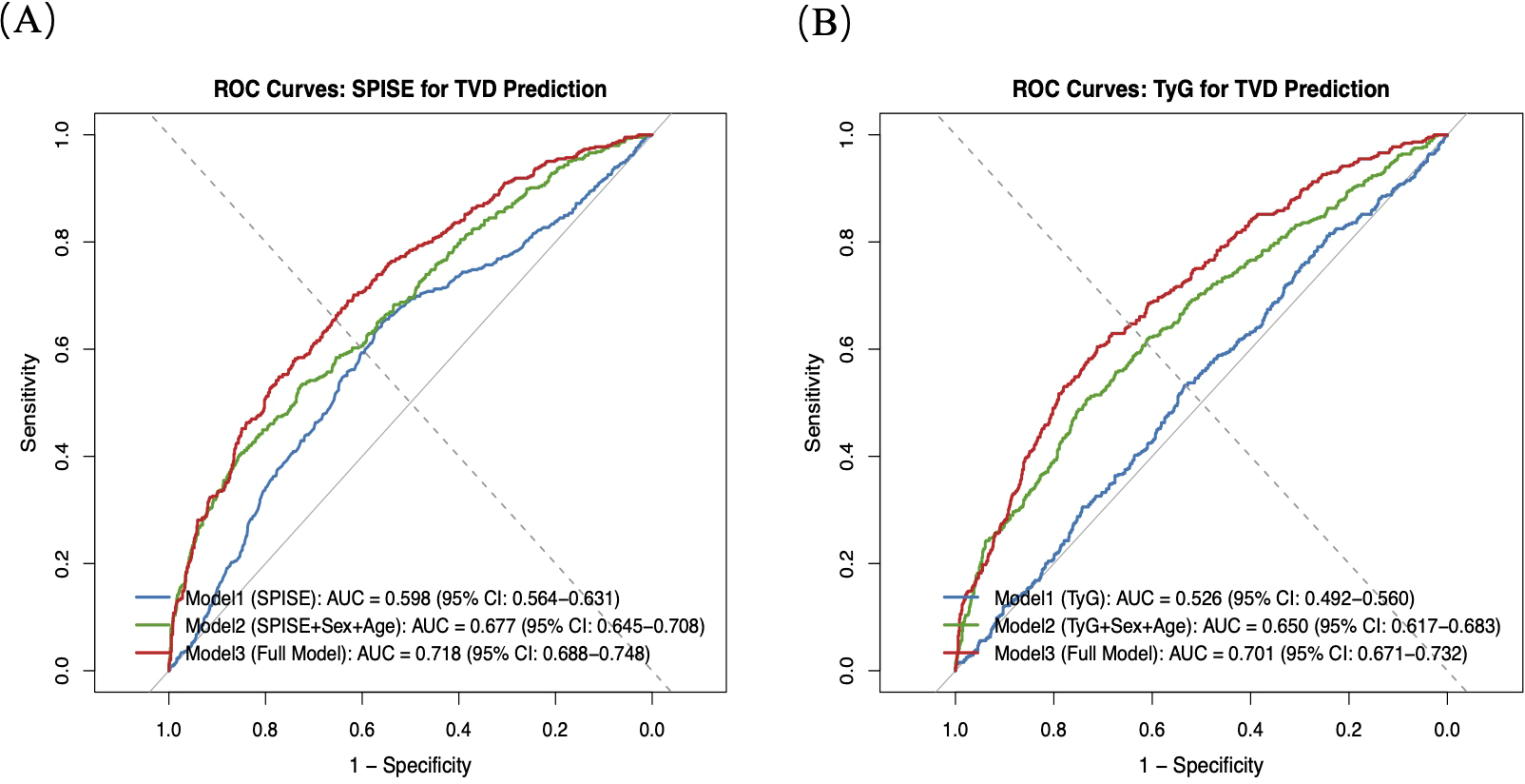
ROC curves for predicting TVD. **(A)** ROC curve for predicting TVD using SPISE. **(B)** ROC curve for predicting TVD using TyG. ROC, receiver operating characteristic; AUC, area under the curve; SPISE, single-point insulin sensitivity estimator; TVD, three-vessel coronary disease; TyG, triglyceride–glucose index.

Receiver operating characteristic (ROC) curve analysis was performed to evaluate and compare the predictive performance of SPISE and the TyG index for TVD, using the area under the curve as the primary metric (**Figure 3**). For SPISE, the AUC values were 0.598 (95% CI: 0.564-0.631) in Model 1, 0.677 (95% CI: 0.645- 0.708) in Model 2, and 0.718 (95% CI: 0.688-0.748) in Model 3. For the TyG index, the corresponding AUC values were 0.526 (95% CI: 0.492-0.560), 0.650 (95% CI: 0.617-0.683), and 0.701 (95% CI: 0.671-0.732), respectively. Across all models, SPISE consistently demonstrated higher predictive performance than the triglyceride glucose index, and the 95% confidence intervals did not overlap. The DeLong test showed that the AUC of SPISE (0.718) was significantly higher than that of TyG (0.701) for predicting TVD events (*P* = 0.012). Bootstrapping analyses further revealed significant incremental value of SPISE over TyG, with a net reclassification improvement (NRI) of 0.035 (*P* = 0.045) and an integrated discrimination improvement (IDI) of 0.020 (*P* < 0.001).

### 3.5 Restricted cubic spline and threshold effect analysis

**Figure 4:**
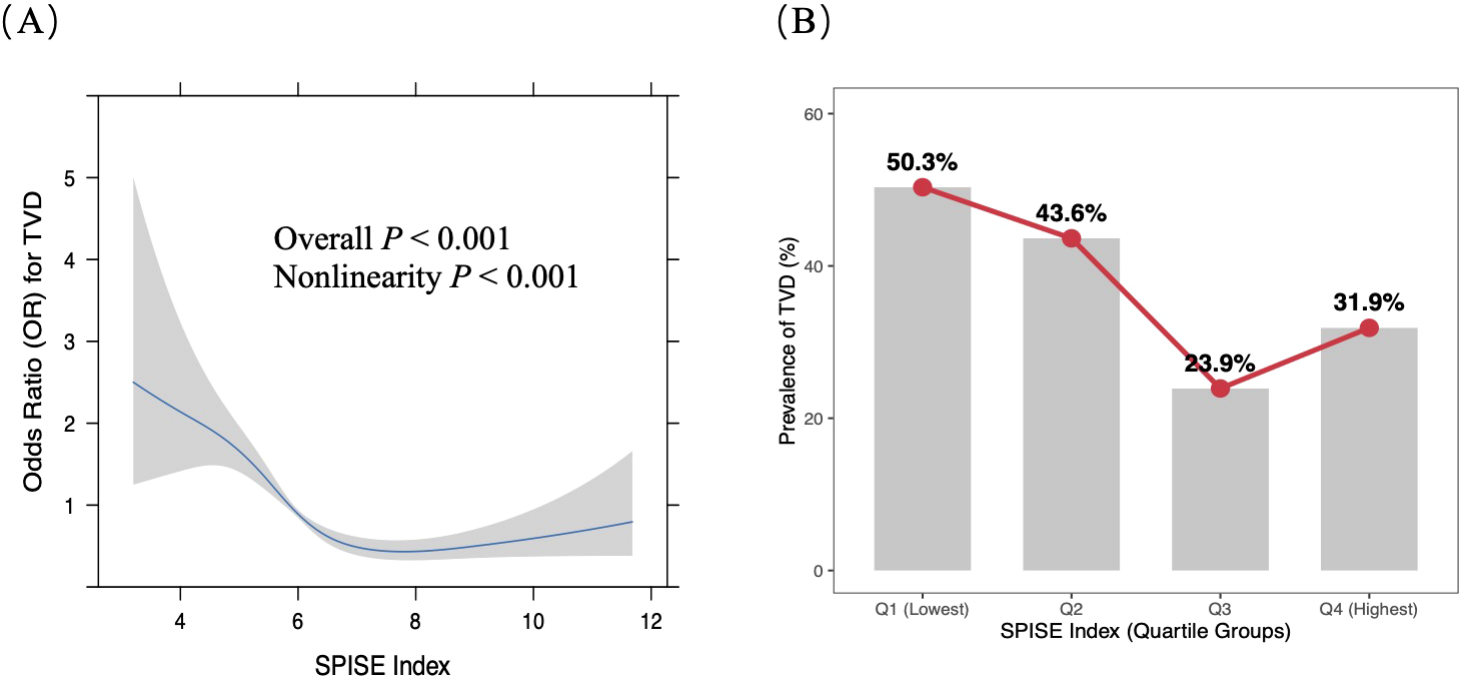
**(A)** Restricted spline curve for the association between SPISE and TVD. **(B)** Quartile bar chart of the association between SPISE and TVD. The blue line represents the OR, with the gray transparent area indicating the 95% confidence interval. SPISE, single-point insulin sensitivity estimator; TVD, three-vessel coronary disease.

After adjustment for potential confounders, including sex, age, smoking, alcohol consumption, hypertension, diabetes mellitus, MI, LVEF, FBG, Cr, Alb, LY, UA, CRP, and HbA1c (**Figure 4**), significant nonlinear relationship was observed between SPISE and TVD (*P* for nonlinearity < 0.001). As SPISE increased, the risk of TVD decreased progressively, and this protective effect tended to plateau beyond a certain level. Segmented regression analysis further identified a threshold, with an inflection point at a SPISE value of 6.76 (*P* < 0.001). A significant inverse association between SPISE and TVD was observed when SPISE was below 6.76 (OR = 0.55, 95% CI: 0.45-0.68), whereas no significant association was detected when SPISE exceeded 6.76. These findings suggest a saturation-type nonlinear relationship between SPISE and TVD risk.

### 3.6 Subgroup analysis

Subgroup and interaction analyses were performed to assess the robustness of the association between SPISE and TVD (**Table 3**). Overall, SPISE was significantly inversely associated with TVD in the entire cohort (OR = 0.75, 95% CI: 0.68-0.83, *P* < 0.001). Significant interactions were observed across subgroups defined by sex, alcohol consumption, age, and BMI (all *P* < 0.05). The inverse association between SPISE and TVD was more pronounced in males, non-drinkers, individuals younger than 65 years, and those with a BMI between 24 and 28 kg/m². No significant interactions were found in subgroups stratified by smoking status, hypertension, or diabetes mellitus (all *P* > 0.05).

**Table 3:** Subgroup analysis of SPISE with TVD.

Variables	n (%)	OR (95% CI)	*P*	*P* for interaction	FDR-adjusted *P*
All patients	1188 (100.00)	0.75 (0.68 ~ 0.83)	<0.001		
Sex				0.013	0.024
Female	416 (35.02)	0.81 (0.69 ~ 0.94)	0.007		
Male	772 (64.98)	0.70 (0.61 ~ 0.80)	<0.001		
Smoke				0.928	0.928
No	802 (67.51)	0.73 (0.64 ~ 0.82)	<0.001		
Yes	386 (32.49)	0.80 (0.67 ~ 0.95)	0.011		
Drink				0.020	0.029
No	1007 (84.76)	0.72 (0.64 ~ 0.80)	<0.001		
Yes	181 (15.24)	0.98 (0.76 ~ 1.27)	0.885		
Diabetes				0.437	0.516
No	814 (68.52)	0.75 (0.66 ~ 0.84)	<0.001		
Yes	374 (31.48)	0.75 (0.62 ~ 0.91)	0.004		
Age group				<0.001	<0.001
<65	626 (52.69)	0.58 (0.48 ~ 0.69)	<0.001		
≥65	562 (47.31)	0.84 (0.74 ~ 0.94)	0.004		
BMI group				<0.001	<0.001
<24	464 (39.06)	1.05 (0.90 ~ 1.23)	0.506		
24–28	522 (43.94)	0.56 (0.42 ~ 0.75)	<0.001		
>28	202 (17.00))	0.89 (0.56–1.42)	0.625		
Hypertension				0.539	0.622
No	493 (41.50)	0.73 (0.62–0.85)	<0.001		
Yes	695 (58.50)	0.77 (0.67–0.88)	<0.001		

OR, odds ratio; CI, confidence interval. *P* for interaction was derived from multiplicative interaction terms. FDR-adjusted *P* values account for multiple comparisons.

## 4 Discussion

A total of 1,188 participants were included in this study, among whom 37.5% (n = 445) were diagnosed with TVD. Multivariable logistic regression analysis demonstrated that SPISE was independently and inversely associated with the risk of TVD, whether analyzed as a continuous or categorical variable. This association remained stable and robust after adjustment for potential confounders. Receiver operating characteristic curve analysis further showed that, compared with the triglyceride glucose index, SPISE exhibited superior predictive performance for TVD risk.

RCS analysis revealed a significant nonlinear association between SPISE and the risk of TVD. Piecewise linear regression further identified an inflection point at a SPISE value of 6.76, providing a clinically relevant and quantifiable threshold that enhances the practical utility of SPISE in risk prediction. Specifically, when SPISE was below 6.76, it was significantly and inversely associated with TVD risk, and its predictive performance was most pronounced in this range. These findings suggest that in individuals with more evident IR, SPISE may serve as a sensitive indicator linking metabolic abnormalities to coronary artery disease severity, thereby facilitating early identification of high-risk patients. In contrast, when SPISE exceeded 6.76, the association with TVD risk became attenuated, reflecting a saturation pattern that is commonly observed in metabolic markers of cardiovascular risk.

Subgroup analyses further indicated that the association between SPISE and TVD varied across different clinical subpopulations. Significant interactions were observed for sex, alcohol consumption, age, and BMI. The predictive value of SPISE was more pronounced in males, non-drinkers, individuals younger than 65 years, and those with a BMI between 24 and 28 kg/m². In contrast, its predictive performance was attenuated in females, individuals who consume alcohol, older adults, and those who were underweight or obese. This heterogeneity may be explained by the underlying pathophysiological differences in IR across these subgroups. In younger and middle-aged populations, metabolic plasticity is relatively preserved, and changes in insulin sensitivity can more sensitively reflect the degree of systemic metabolic disturbance, thereby showing a closer association with the severity of coronary artery disease (21, 22). In contrast, in older individuals, age-related processes such as vascular aging and structural remodeling tend to predominate, which may attenuate the contribution of metabolic status to coronary lesion severity. Among individuals with a BMI of 24 to 28 kg/m², those with higher BMI may exhibit a broader spectrum of insulin sensitivity and resistance (23), SPISE may more effectively capture the impact of metabolic abnormalities on coronary artery disease risk. In addition, alcohol consumption may interfere with lipid metabolism and insulin signaling pathways (24, 25), thereby obscuring the intrinsic relationship between SPISE and coronary artery disease. This may explain why a stable association was observed primarily in non-drinkers. The reduced predictive performance of SPISE in females, drinkers, older individuals, and those who are underweight or obese may be attributed to the influence of other factors affecting metabolic status, such as hormonal variation, alcohol-related metabolic interference, and nutritional status (26–28), which may weaken the link between IR and CAD. Notably, no interaction was observed in subgroups stratified by smoking status, hypertension, and diabetes. This suggests that, as an indicator of IR, SPISE retains a certain degree of independence in predicting TVD risk and is not substantially confounded by these traditional cardiovascular risk factors. Heterogeneity was observed in subgroup analyses stratified by sex, alcohol consumption, age, and BMI. In addition, the small sample size of the alcohol-drinking subgroup (181 participants, 15.2%) may limit statistical power. These findings indicate that the subgroup analyses are exploratory in nature, and the corresponding results should be interpreted with caution.

A study incorporating Mendelian randomization analysis has demonstrated that IR is associated with hypertension and a range of cardiovascular diseases (29). Other studies have shown that even in the absence of diabetes, IR can serve as a marker for assessing the severity of coronary artery disease and for clinical risk stratification (30, 31). In this context, practical and reliable surrogate markers of IR are of particular importance. The TyG index, a simple and emerging surrogate of IR, has been shown to have robust predictive value in patients with stable CAD, acute coronary syndromes, myocardial infarction, non-obstructive coronary artery disease, and TVD (32–34). In the present study, receiver operating characteristic curve analysis further demonstrated that SPISE, as a novel indicator of IR, exhibited superior predictive performance for TVD compared with the TyG index, with an area under the curve of 0.718 versus 0.701. The superior predictive ability of SPISE may be attributed to its formula design and its more comprehensive reflection of metabolic status. Specifically, SPISE incorporates HDL-C, TG, and BMI, thereby capturing key dimensions related to IR and atherosclerosis, including lipid metabolism and adiposity. This integrative approach enables SPISE to more effectively reflect the systemic metabolic disturbances associated with the development of TVD. In contrast, the TyG index includes only TG and FBG, without accounting for body composition or specific lipid subfractions, which may limit its ability to fully characterize the metabolic abnormalities underlying TVD.

Although the HEC remains the reference standard for assessing insulin sensitivity, its invasive nature, procedural complexity, high cost, and requirement for specialized equipment and expertise restrict its use primarily to small-scale research settings. While SPISE cannot provide the precise quantitative assessment of IR achieved by this method, it offers distinct advantages in terms of being noninvasive, simple, cost-effective, and readily obtainable in routine clinical practice. These features make SPISE particularly suitable for large-scale screening of IR and for cardiovascular risk prediction, highlighting its substantial clinical applicability and potential for widespread use. Overall, compared with TyG, SPISE demonstrated superior predictive performance for TVD. In comparison with the HEC, SPISE is more practical for routine clinical use while maintaining scientific validity. As an IR related index derived from commonly available clinical parameters, SPISE is well suited for use in healthcare settings lacking specialized testing facilities and in large-scale clinical studies, highlighting its substantial translational potential.

### 4.1 Limitations

Several limitations should be acknowledged. First, due to the retrospective design and the scope of routine clinical measurements in the cohort, complete data on interleukin-6 (IL-6) and diabetes severity and duration were unavailable. These factors were not included in confounding adjustment, potentially introducing residual confounding into the association analysis between SPISE and TVD. Strict exclusion criteria resulted in a highly selected study sample, all patients underwent coronary angiography, indicating that the cohort represented a prescreened high-risk cardiovascular population rather than an unselected general population. Accordingly, the generalizability of our findings may be limited, and the observed association may not be directly extrapolated to broader clinical populations. Second, this study is a single-center, cross-sectional analysis in which SPISE and TVD were assessed concurrently, precluding causal inference and leaving the possibility of reverse causation. These findings represent exploratory results only and lack external validation across diverse populations as well as standardized clinical reference values. Future prospective, multicenter, longitudinal studies enrolling Western and multiethnic populations are therefore warranted. Third, TVD was defined solely based on ≥50% angiographic stenosis, without incorporating functional significance or lesion complexity. Important assessments such as FFR/iFR and the SYNTAX score were not available, which may lead to an oversimplified characterization of TVD and limit the interpretation of its association with SPISE. Notably, the cutoff value identified in this study warrants highly cautious interpretation. At present, this threshold should not be regarded as a clinically applicable standard, as it represents a preliminary exploratory result that currently lacks sufficient biological evidence and independent validation to justify direct clinical implementation. Furthermore, the clinical applicability of our findings is constrained by the absence of established risk scoring systems, structured clinical decision-making frameworks, and validated clinical cutoff values.

In conclusion, SPISE is inversely associated with the risk of TVD. Given its simplicity and accessibility, SPISE may serve as a valuable clinical indicator for predicting TVD and may facilitate more individualized strategies for the prevention and management of cardiovascular disease. However, large-scale randomized controlled studies are still required to further evaluate its prognostic value in patients with TVD.

## Data Availability

The original contributions presented in the study are included in the article/supplementary material. Further inquiries can be directed to the corresponding authors.
